# The Role of Systems Vaccinology in Understanding the Immune Defects to Vaccination in Solid Organ Transplant Recipients

**DOI:** 10.3389/fimmu.2020.582201

**Published:** 2020-11-25

**Authors:** Nicholas Scanlon, Youssef Saklawi, Nadine Rouphael

**Affiliations:** ^1^ Department of Medicine, School of Medicine, Emory University, Atlanta, GA, United States; ^2^ The Hope Clinic of the Emory Vaccine Center, Division of Infectious Diseases, Emory University, Decatur, GA, United States

**Keywords:** vaccine, transplant, systems biology, systems immunology, systems vaccinology, immunocompromised, immunization

## Abstract

Solid organ transplant recipients (SOTRs) are at increased risk for many infections, whether viral, bacterial, or fungal, due to immunosuppressive therapy to prevent organ rejection. The same immune defects that render transplanted patients susceptible to infection dampen their immune response to vaccination. Therefore, it is vital to identify immune defects to vaccination in transplant recipients and methods to obviate them. These methods can include alternative vaccine composition, dosage, adjuvants, route of administration, timing, and re-vaccination strategies. Systems biology is a relatively new field of study, which utilizes high throughput means to better understand biological systems and predict outcomes. Systems biology approaches have been used to help obtain a global picture of immune responses to infections and vaccination (i.e. systems vaccinology), but little work has been done to use systems biology to improve vaccine efficacy in immunocompromised patients, particularly SOTRs, thus far. Systems vaccinology approaches may hold key insights to vaccination in this vulnerable population.

## Introduction

Systems biology was described by Alan Aderem as a “comprehensive quantitative analysis of the manner in which all components of the biological system interact functionally over time and space that is executed by an interdisciplinary team of investigators” (1). Systems biology uses high throughput “-omics” technologies to investigate the structure and dynamics of the entire system to predict outcomes (2). In a systems biology approach, the system is perturbed as a result of an infection or immunization; genes, proteins, lipids, sugars, and molecular pathways are monitored; data are collected, analyzed, and integrated; and mathematical models are formulated to describe or predict how the system may respond to specific perturbations (3). Systems immunology takes advantage of the many ways the immune system can be manipulated to better understand signaling pathways in the immune system and how the innate and adaptive immune systems interact to protect against various pathogens (4). When applied to vaccines, systems biology can give us a better understanding of the immune system in general and the optimal immunological response needed for protection. Systems vaccinology utilizes immunization as a way to probe the immune system in a synchronized fashion and effects on the immune system are studied at various timepoints after. This approach can identify early signatures associated with protection, separate vaccinees into responders and non-responders, and can reveal important mechanistic insights through translational human vaccine trials to aid in the expedited design of future vaccines to disease where no effective vaccine exists (e.g. HIV) or to protect vulnerable populations (e.g. elderly, HIV infected and SOTRs) (5). While a number of studies have implemented a systems vaccinology approach to better understand the immune response to various immunizations, very little is published regarding systems vaccinology in immunocompromised patients, particularly solid organ transplant recipients (SOTRs). It is well known that individuals are at risk for infection following solid organ transplant, but little is known about the immune defects to vaccination in these patients. Systems vaccinology has allowed us to have a better understanding of how successful vaccines induce adequate immune responses in healthy subjects and how immune defects are uncovered in other vulnerable populations (e.g., the elderly). This blueprint may offer a personalized approach to vaccination in SOTR.

## Systems Vaccinology in Immunocompetent Hosts

Early studies in systems vaccinology have used a systems biology approach to obtain a global picture of the molecular networks driving vaccine immunity in immunocompetent hosts as opposed to immunocompromised hosts. The yellow fever vaccine 17D, trivalent inactivated (TIV) and live attenuated (LAIV) influenza vaccines, and meningococcal quadrivalent polysaccharide (MPSV4) and meningococcal quadrivalent conjugate vaccines (MCV4) were among the first to be studied in-depth using this approach. The hepatitis B virus (HBV) vaccine has also been studied utilizing a systems biology approach.

### Yellow Fever Vaccine 17D

The first studies to utilize a systems biology approach analyzed the immune responses to the yellow fever vaccine 17D (YF-17D), a live attenuated vaccine highly effective with close to 90% rate of protection (6, 7). The study noted a difference in the magnitude of neutralizing antibody titers and antigen-specific cytotoxic T lymphocyte (CTL) responses at days 15 and 60 between different individuals. Two genes were predictive up to 90% of a high magnitude adaptive immune response: *EIF2AK4* (a critical player in the integrated stress response, resulting in a shutdown of translation of most proteins in the cell) and *TNFRSF17* (which encodes the receptor for B-cell growth factor BLyS-BAFF and plays a role in the differentiation of plasma cells) (7). The authors were able to predict the immunogenicity of YF-17D with innate immune signatures. Thereby, the study laid the groundwork for using a systems biology approach to predict the magnitude of the adaptive immune response to vaccine early on.

### Trivalent Inactivated (TIV) and Live Attenuated (LAIV) Influenza Vaccine

Nakaya et al. in 2011 extended a systems biology approach to investigate the innate and adaptive immune responses to the TIV and live attenuated influenza vaccines in humans. Their objective was to determine whether similar signatures, which were predictive of the adaptive immune response in YF-17D were present with TIV and LAIV. They found that LAIV induced a robust type I IFN antiviral transcriptomic signatures. TIV also induced the expression of genes encoding type I IFNs as well as pro-inflammatory mediators and genes involved in the innate sensing of viruses 1–3 days after vaccination and then genes such as *TNRSF17* and others known to be involved in the differentiation of plasmablasts; these correlated well with the magnitude of hemagglutinin titers 28 days after immunization. Another gene, calmodulin-dependent protein kinase IV (*CaMKIV)* was shown to have an expression profile inversely proportional to later antibody titers. LAIV did not induce as robust of an antibody response as TIV. Ultimately, the clinical effectiveness of these two vaccines is known to be similar despite the difference in antibody response. The authors suggested the similar clinical effectiveness may be related to the hypothesized mechanism by which LAIV primes immune cells in the nasal mucosa, which then circulate in the blood to activate other immune cells (8). Delivery method may play an important role in vaccine efficacy. The Human Immunology Project Consortium (HIPC) and the Center for Human Immunology were able to identify transcriptional signatures predictive of response to influenza vaccination. They showed the presence of inflammatory gene signatures was associated with more robust antibody responses in younger individuals, but worse antibody responses in older individuals (9). Ultimately, these studies confirmed that predicting vaccine responses through a systems biology approach was possible in the context of influenza and that baseline immunological status is a potential mechanism by which to understand poor vaccination outcomes in older individuals.

### Meningococcal Quadrivalent Polysaccharide Vaccine (MPSV4) and Meningococcal Quadrivalent Conjugate Vaccine (MCV4)

Another study by Li et al. in 2014, utilized a systems vaccinology approach to investigate the immune response to meningococcal polysaccharide (MPSV4) and meningococcal conjugate vaccine (MCV4) as it compares with that of YF-17D, TIV, and LAIV. Both MPSV4 and MCV4 are capable of inducing high antibody titers post-vaccination, but MPSV4 is thought to induce T-cell independent antibody responses, resulting in waning humoral immunity and memory. The authors analyzed data by merging 32,000 peripheral blood mononuclear cell (PBMC) gene expression profiles from 540 published studies and were able to identify 334 different blood transcriptome modules (BTMs) from existing transcriptomic data in public repositories. The study revealed three distinctive transcriptomic programs, which could potentially be used to predict vaccine efficacy. One transcriptomic program was a protein recall response that correlated with the antibody response to TIV and a portion of MCV4. Another transcriptomic program was a primary viral response elicited by YF-17D. The final transcriptomic program was an anti-polysaccharide signature induced by the polysaccharide portions of MCV4 and MPSV4 (10).

### Hepatitis B Virus (HBV) Vaccine

In 2016, Fourati et al. identified transcriptomic patterns associated with aging and correlated these transcriptomic modules with biological pathways after HBV vaccination. An aggregate score depicting age-related transcriptomic changes (BioAge signature), a surrogate for B-cell activation, was shown to predict the response to the HBV vaccine with a 60% accuracy. Higher levels of baseline memory B cells and CD4+ T cells were associated with a sufficient immune response to vaccination. Additionally, 15 gene expression patterns related to inflammation and interferon signaling pathways are significantly different between vaccine responders and non-responders (11). Such immunologic patterns may be used in addition to age and patient demographics to account for baseline heterogeneity when conducting vaccine clinical trials; leading to more personalized vaccine research. A systems biology approach has also been undertaken to evaluate new adjuvants for the HBV vaccine (12).

## Systems Vaccinology in Vulnerable Populations

Systems biology approaches have emerged to assess vaccination in vulnerable populations such as in people living with HIV (13) while vaccination in other vulnerable populations such as neonates has yet be studied using a systems biology approach; these populations may benefit as well. Another vulnerable population in regard to infection and suboptimal response to vaccination is the elderly which constitute 16% of the US population. More than 90% of seasonal influenza-related deaths occur among people over 60 years of age (14). Stressing the importance of better understanding immunosenescence to design more effective vaccines for a subpopulation most affected by influenza mortality (15). Nakaya et al. applied a systems biology approach comparing the immune responses to influenza vaccine in young adults and elderly across many seasons (16). The fold changes in hemagglutination inhibition titers (HAI) were statistically higher in the younger versus the older group revealing a correlation of decreased antibody responses to influenza vaccine with age. When compared to the younger group, the older group exhibited a diminished B cell and an increased frequency and activation of NK cell responses after vaccination as well as an enhanced monocyte response pre and post vaccination. There was also a difference in expressed genes between the two groups mostly noted one day after vaccination with a greater number of both up- and downregulated genes observed in the younger group. While both groups had similar temporal expression profiles by clusters, the magnitude of the expression of interferon-related genes was also higher in the younger group. Studies have shown methylation and the transcriptome may play a role in and predict humoral immunity. One analysis looked at how methylation affects the expression of genes known to play a role in humoral immunity (17). Gene signatures associated with influenza-specific memory B-cell responses were identified by transcriptome-wide profiling of peripheral blood mononuclear cells (PBMCs) (18). The suboptimal vaccine immune responses in the elderly could be improved by the use of FDA approved seasonal influenza vaccine products such as adjuvant (MF59 oil in water adjuvant) (19) or high-dose vaccines (20) (with 60 mcg of hemagglutinin per strain, the equivalent of four times the current amount of HA in seasonal influenza vaccines). Immunosenescence has been a key focus of systems vaccinology and can likely provide insight into the immune defects to vaccination SOTRs possess.

## Immunosuppression and Infection in SOTRs

Historically, acute rejection was common after transplant, but over the years, T cell-mediated allo-immune responses have been targeted for most immunosuppression drug development in transplantation (21). In SOTRs, the survival of the patient and graft rely on lifelong modulation of the immune system. Immunosuppressive agents are given perioperatively to prevent allograft rejection. This induction therapy serves to deplete T cells, thereby reducing acute rejection rates and enhancing allograft survival. Maintenance immunosuppression consists of multiple medications, which target various aspects of the immune response. Most transplant centers use a triple-drug regiment including the second-generation calcineurin inhibitor (CNI) tacrolimus, the antiproliferative agent mycophenolic acid, and a corticosteroid; rapamycin-based therapies are sometimes used instead of calcineurin-based therapies to preserve long-term renal function (22). Calcineurin inhibitors such as cyclosporine and tacrolimus work by reducing interleukin-2 (IL-2) production and IL-2 receptor expression, which leads to decreased T-cell activation (23). Inhibitors of mammalian target of rapamycin (mTOR) such as sirolimus and everolimus work later in the cell cycle to prevent IL-2-mediated T-cell proliferation and can act synergistically with cyclosporine and tacrolimus (22). Mycophenolic acids such as mycophenolate mofetil (MMF) act by interfering with purine synthesis to selectively inhibit T and B-lymphocyte proliferation (22). Finally, corticosteroids act through multiple mechanisms, including inhibition of interleukins in macrophages and monocytes, inhibition of the expression of cytokines, and inducing programmed cell death of T cells (22). The effects of corticosteroids on the human immunome have also been described. One study showed that systemic glucocorticoids down-regulate inflammatory cytokine levels in humans and that there was an inhibitory effect on transcription modules associated with inflammation at early time points (24). Their study suggested that anti-inflammatory effects of glucocorticoids are a result of modulation of mRNA levels (24).

The immunome of recipients often determines the degree of response to vaccination. Models based on a small subset of immune cells may be sufficient to predict immune reactivity, whether to vaccines or auto-immune disease flares (25, 26). As aforementioned, immunosuppressive agents strongly alter the immune landscape, and would predictably alter the response to vaccination. Multiple studies have shown that vaccines are less immunogenic in SOTRs (27), and some studies have demonstrated a direct effect of particular immunosuppressive agents used in this population. In fact, it has been shown that MMF has a dose-dependent response where higher doses, particularly greater than or equal to 2 grams daily were associated with lower seroconversion rates to influenza vaccination (28). Additionally, m-TOR inhibitors were shown to decrease antibody response to the pandemic H1N1-2009 vaccination (29). Another study showed that less seroprotection for influenza after vaccination was achieved in renal transplant patients who had received tacrolimus-based regimens compared with healthy controls (30). Corticosteroids and other immunosuppressive agents used in this population were associated with significantly impaired response to the 23-valent pneumococcal polysaccharide vaccination (23vPPV) (31). Liver transplant patients have been shown to infrequently benefit from hepatitis B vaccination as one study showed only 20% of patients developed measurable anti-HBs in response to vaccination whereas seroconversion rates in healthy adults are greater than 90% (32).

## Systems Vaccinology in SOTRs

Most studies related to vaccinology in SOTR have looked mostly at serologic markers to assess vaccine immunogenicity. Seroprotection and seroconversion in SOTRs in response to influenza vaccination has varied between 15–93% (33). SOTRs may have high titers of cross-reactive antibodies due to frequent yearly influenza vaccination, which may explain in part the low seroconversion rate among this population (34). Similarly, vaccine-induced serologic immunity to the measles vaccine in SOTR pediatric population was shown to wane over time, along with impaired measles-specific B-cell distribution and immune senescence (35). Few studies have tried to assess the association between the antibody responses and the cellular, and cytokine responses. A study in lung transplant recipients showed an impaired cell-mediated immune response to influenza vaccination by assessing granzyme B and interleukin production (36). Different studies have alluded to an association between humoral and cellular responses (36–38). Some are summarized in **Table 1**. There is a paucity of data surrounding vaccine-induced immunity in SOTRs relevant to the innate immunity and systems biology in general, so it is vital that more studies investigate this area.

**Table 1:** Summation of studies utilizing a systems biology approach in SOTRs.

First Author	Year	Title	Vaccine	Transplant	Data Assessed	Findings
Soesman (39)	2000	Efficacy of influenza vaccination in adult liver transplant recipients.	Influenza	Liver	Humoral and Cellular Immunity	Postvaccination virus-specific T cell proliferation lower than controls (not statistically significant).
Mazzone (36)	2004	Cell-mediated immune response to influenza vaccination in lung transplant recipients	Influenza	Lung	Humoral and Cellular Immunity	Virus-specific responses (Granzyme B & cytokine production) impaired, while antibody response maintained.
Ballet (38)	2006	Humoral and cellular responses to influenza vaccination in human recipients naturally tolerant to a kidney allograft	Influenza	Kidney	Humoral and Cellular Immunity	Comparable humoral and cellular responses to vaccination in SOTRs after cessation of immunosuppressive therapy.
Candon (37)	2009	Humoral and cellular immune responses after influenza vaccination in kidney transplant recipients	Influenza	Kidney	Humoral and Cellular Immunity	Increase in interferon producing T cells post-vaccination in SOTRs and healthy controls, no association with humoral response.
Cagigi (13)	2013	Premature ageing of the immune system relates to increased anti-lymphocyte antibodies (ALA) after an immunization in HIV-1-infected and kidney-transplanted patients	Influenza	Kidney	B-cell biomarkers	Diminished levels of interleukin-21 and interleukin-21 receptor expression in SOTRs postvaccination, along with higher levels of mature activation of B cells and double negative B cells compared to healthy controls.
Rinaldi (34)	2014	B-sides serologic markers of immunogenicity in kidney transplanted patients: report from 2012-2013 flu vaccination experience	Influenza	Kidney	B-cell biomarkers	Influenza specific memory B-cell postvaccination in SOTR similar to healthy controls independent of seroconversion.
Rocca (35)	2016	Waning of vaccine-induced immunity to measles in kidney transplanted children	Measles	Kidney	B-cell biomarkers	Waning of antibody and B-cell responses to measles in pediatric patients Seroprotection depends on immune status at vaccination.

Immunosuppressive agents dramatically reduce the risk of rejection in transplanted patients while at the same time increasing the patient’s risk for opportunistic infections. Thus, general strategies such as vaccination, universal prophylaxis, and preemptive therapies are used to mitigate the risk of infection. Current guidelines recommend the need for immunization be evaluated, and if possible completed, before transplantation as vaccinations may not be as immunogenic after transplantation (40, 41). Immunosuppressive regimens vary between organ transplants, and some organs like the heart require more aggressive and long-term immunosuppression. Cases of clinical operational tolerance have been described in kidney and liver transplants, but rarely in pancreatic, intestinal, heart, or lung transplants (42). This suggests a varied immune landscape among SOTRs, which requires further characterization through systems biology studies. A better immunologic understanding behind a tailored preventative approach through immunization is needed for SOTRs.

## Future Directions for Systems Vaccinology

While studies have utilized systems vaccinology to help better understand and predict how well a vaccine will work in the elderly and improve that response, it is vital that we use systems vaccinology to improve vaccines for immunosuppressed populations such as SOTRs.

### Vaccine Design in Solid Organ Transplant

Various vaccination strategies have been discussed in the literature to combat the decreased immunogenicity of vaccines in SOTRs particularly to influenza vaccines (43, 44).

#### Adjuvants

Adjuvants enhance the immune response to vaccine antigen by nonspecifically stimulating cells of the innate immune system; however, they represent a diverse range of materials from small synthetic molecules to heterogeneous extracts of natural products. Aluminum salts (Alum) have historically been the most common adjuvant included in vaccines. Over the past few decades, vaccines have been formulated with novel adjuvants; these include vaccines against HBV, HPV, influenza, and VZV (45). Some have been studied in the elderly (46) and transplant populations (47) to determine efficacy and safety. Adjuvants work by delivering a localized activation signal to the innate immune system, thereby promoting antigen-specific adaptive immunity. Comparative studies of different adjuvants are sparse, and the mechanism of action is poorly understood (48). Future studies elucidating such knowledge can improve vaccine design and implementation. A systems biology approach can be utilized to select the ideal antigen/adjuvant combination through an evidence-based approach allowing for the more expedited development of effective adjuvanted vaccines in SOTRs. Additionally, a systems approach may represent a better technique for risk surveillance and mitigation through better prediction of immune reactivity and potential transplant rejection (49).

However, adjuvants could represent a safety concern in transplant patients. One study, which compared an adjuvanted influenza vaccine containing an oil-in-water emulsion adjuvant (MF59) to a nonadjuvanted formulation showed comparable immunogenicity and seroprotection; a subgroup analysis of the 18–64-year-old group showed greater seroconversion rates in the adjuvanted vaccine group. There was no increase in Human Leukocyte Antigen (HLA) alloantibodies in those receiving the adjuvanted vaccine, suggesting it was safe in these patients (50).

#### Timing of Vaccination

If vaccinations are not given before transplant, current guidelines recommend transplant patients receive vaccinations approximately 3–6 months after transplantation when baseline immunosuppression levels are obtained; however, there is little data regarding the ideal timing of vaccination post-transplant (41). One study of influenza vaccination showed that those less than 6 months after transplant and on daily MMF and prednisone were at risk for poor vaccine response largely due to the intensity of immunosuppression in the first 6 months (28). A study showed in liver transplant patients that only 14% responded to influenza vaccine 4 months post-transplant, 67% seroconverted at 4–12 months, and 86% after 12 months (51). This supports the current recommendation for influenza vaccination administration 3–6 months post-transplant when patients are on less intense immunosuppressive regimens (41). In contrast to these studies, a multicenter prospective cohort study in adult SOTRs looked at influenza vaccination over four influenza seasons from 2009–2013 (52). After adjusting for confounders, they found that seroprotection was similar in those vaccinated within 6 months of transplantation and those vaccinated more than 6 months after transplantation (52). Our group is currently investigating the optimal timing of the AS01-adjuvanted varicella zoster virus subunit (HZ/su) vaccine in kidney transplant recipients. (NCT 03993717) Systems biology may also be aimed to detect time points of optimal immune activation, potentially leading to personalized vaccine administration schedules per real-time immune status of patients (26).

#### Vaccine Dosing

Another vaccine strategy that could increase immunogenicity in SOTRs is increasing vaccine dosing. A recent RCT conducted by the TRANSGRIPE 1–2 Study Group used a booster dose of inactivated influenza vaccine 5 weeks from the original dose in SOTRs after one month of transplant. It showed that this was associated with higher short-term seroconversion rates in per-protocol analysis, but not in the intention to treat group; seroprotection as 10 weeks was also higher in the booster group with the number needed to treat being less than 10 (53). Another study looked at two doses of the influenza A/H1N1 (2009) pandemic vaccine in kidney transplant patients and showed this provided significantly improved seroprotection (54). A systematic literature review and meta-analysis, which pooled data from multiple influenza vaccination studies showed no enhanced immunogenicity of a booster dose of influenza vaccine in renal transplant patients (55). More recently, a double-blind, randomized trial showed that high-dose influenza vaccine (including 3 vaccine strains) had significantly improved immunogenicity and similar safety in SOTRs (56).

#### Delivery Method

Vaccines can be delivered intramuscularly, intradermally, subcutaneously, orally, and intranasally; the latter two routes are not used in SOTRs as they are live-attenuated vaccines. Most commonly, vaccines have been administered intramuscularly and subcutaneously; however, intradermal vaccines are thought to improve immunogenicity by increasing exposure of the antigen to antigen presenting cells such as dendritic cells. A 2011 cohort study of 85 lung transplant recipients receiving the seasonal 2008–9 inactivated influenza vaccination showed a poor response in both the 6 µg intradermal group and the 15 µg intramuscular group (57). Later, a study looked at higher doses, 18 µg intradermal and 15 µg intramuscular and showed improved, but similar immunogenicity in lung transplant patients (28). Another novel delivery route is microneedle patch technology that can be self-administered, thermostable and leaves no sharp waste (58). This technology has been studied as a mechanism to administer the influenza vaccine (59). Since microneedle patch targets the superficial layers of the skin rich in dendritic cells it may offer antigen sparing and better antigen delivery ultimately leading to an enhanced immune response particularly in vulnerable and immunocompromised populations (60).

## Discussion

SOTRs represent a vulnerable population when it comes to infection, and vaccination remains one of the most effective means to prevent infection in this population. While current guidelines recommend vaccination prior to transplant, there is significant variability in implementation of this recommendation in SOTRs. Furthermore, vaccination in SOTRs, is known to produce suboptimal immune responses compared with immunocompetent individuals. Most transplant centers initiate vaccination 3–6 months post-transplant, at the time immunosuppression levels are obtained, for those who have not completed all vaccinations prior to transplant. Influenza vaccinations can be given as early as 1 month after transplant. It is recommended that serologic response be obtained a minimum of 4 weeks after vaccination to document seroconversion based on protective titers in established literature. However, serology is not necessarily an accurate measure of immunity, particularly post-transplant (41). Furthermore, decreased vaccine-specific immune responses and waning titers after transplant are well-documented (61). Consequently, vaccination should not follow a “one-size fits all” model, particularly in immunosuppressed and SOTRs. It is important that we focus on the rational design and implementation of efficacious vaccinations as well as evaluation of their immunogenicity in this vulnerable population. There are many research questions that we must ask when considering optimal vaccination strategies in SOTRs. What is the optimal timing of vaccination in SOTRs in relation to immunosuppression? Are adjuvants necessary to boost the immune response in SOTRs, and is the use of adjuvants safe in this population? Do SOTRs need a higher dose, or repeated vaccination in contrast to immunocompetent individuals? And what is the ideal delivery method for vaccinations in this population? Innovative systems biology approaches can be utilized to model critical determinants to predict vaccine success, better characterization of SOTR immune profile, better assessment of patient heterogeneity in research, vaccine response, and prediction of side effects. These systems biology approaches can help us to answer each of the aforementioned questions and determine the optimal timing, potential need for adjuvants, dosing strategy, and delivery method in this unique population. We highly recommend adapting systems biology approaches to optimize vaccination strategies in SOTRs.

## Author Contributions

All authors contributed to the article and approved the submitted version.

## Funding

This work was supposed by the NIH R38 Stimulating Access to Research in Residency (StARR) grant (5R38AI140299-02).

## Conflict of Interest

NR received research funding unrelated to this paper from Sanofi-Pasteur, Pfizer, Quidel, Eli Lilly, and Merck.

The remaining authors declare that the research was conducted in the absence of any commercial or financial relationships that could be construed as a potential conflict of interest.
